# Cue-specific sexual avoidance, metacognitive beliefs, and sexual functioning in women with lifelong vaginismus: a cross-sectional case–control study

**DOI:** 10.1186/s12905-026-04761-z

**Published:** 2026-08-03

**Authors:** Gülin Özdamar Ünal, Sinay Önen, Memduha Aydın

**Affiliations:** 1 Department of Psychiatry, Süleyman Demirel University Faculty of Medicine, Cünür Mah. Doğu Yerleşkesi, Merkez, Isparta, 32200 Türkiye; 2 Department of Psychiatry, University of Health Sciences Bursa Yüksek İhtisas Training and Research Hospital, Kurtuluş Mah. Halide Edip Adıvar Cad. No:18, Nilüfer, Bursa, 16285 Türkiye; 3 Department of Psychiatry, Selçuk University Faculty of Medicine, Ardıçlı Mah. Celal Bayar Cad. No:313, Selçuklu, Konya, 42250 Türkiye

**Keywords:** Vaginismus, Genito-pelvic pain/penetration disorder (GPPPD), Metacognition, Sexual avoidance, Sexual functioning, Women’s sexual health

## Abstract

**Background:**

Avoidance is a key maintaining factor in lifelong vaginismus and may extend beyond penetration attempts to broader sexual cues. Metacognitive beliefs, particularly beliefs about the usefulness of worry and the uncontrollability and danger of thoughts, may shape threat appraisal and coping responses in sexual contexts. We characterized clinician-elicited sex-related avoidance behaviors in lifelong vaginismus and examined how avoidance and metacognitive beliefs relate to sexual functioning beyond anxiety and depressive symptoms.

**Methods:**

This cross-sectional case–control study was conducted in three outpatient clinics in Türkiye. Participants were 64 women evaluated for lifelong vaginismus and 30 controls. Sex-related avoidance over the past 6 months was assessed using five standardized yes/no clinician-elicited items; endorsing ≥ 1 item defined the avoidant subgroup. Participants completed ASEX (Arizona Sexual Experiences Scale), GRISS (Golombok-Rust Sexual Satisfaction Scale), MCQ-30 (Metacognitions Questionnaire-30), Beck Depression Inventory (BDI), and Beck Anxiety Inventory (BAI). Between-group comparisons examined sexual outcomes and metacognitive beliefs across groups, and hierarchical regression models examined whether MCQ-30 subscales accounted for additional variance in ASEX and GRISS scores beyond BDI and BAI.

**Results:**

Thirty of 64 women with vaginismus (46.9%) were classified as avoidant. The avoidant subgroup showed poorer ASEX and GRISS scores than the non-avoidant subgroup and controls (all adjusted pairwise *p* < 0.05). Women with vaginismus showed greater endorsement of dysfunctional metacognitive beliefs than controls. However, the avoidant and non-avoidant vaginismus subgroups did not differ on MCQ-30 indices. In logistic regression, adding three theoretically selected MCQ-30 subscales did not significantly improve model fit beyond BDI and BAI (Δχ²(3) = 3.84, *p* = 0.279). Adding MCQ-30 subscales accounted for an additional 19% of variance in ASEX-total scores (R²=0.13 to 0.32) and an additional 21% of variance in GRISS-total scores (R²=0.05 to 0.26). Negative beliefs about uncontrollability and danger showed the strongest associations with higher ASEX-total (β = 0.52, *p* = 0.004) and GRISS-total (β = 0.58, *p* = 0.002) scores.

**Conclusions:**

Sex-related cue avoidance beyond penetration attempts was endorsed by nearly half of women with lifelong vaginismus and marks poorer sexual functioning. Dysfunctional metacognitive beliefs—particularly beliefs about uncontrollability and danger—relate to sexual impairment beyond anxiety and depressive symptoms, supporting brief screening for avoided cues and metacognitive targets in clinical assessment.

**Supplementary Information:**

The online version contains supplementary material available at https://doi.org/10.1186/s12905-026-04761-z.

## Introduction

Lifelong vaginismus is characterized by persistent difficulty with vaginal penetration accompanied by marked fear and avoidance, as well as pelvic floor muscle tightening [1, 2]. Although DSM-5 subsumes vaginismus and dyspareunia under Genito-Pelvic Pain/Penetration Disorder (GPPPD), the term lifelong vaginismus is used to denote a primary penetration-related presentation characterized by marked fear-avoidance and pelvic floor tightening in the context of attempted vaginal penetration [3]. Crucially, irrespective of nosological labeling, vaginismus is linked to pronounced impairment in sexual functioning, relationship well-being, and quality of life [4, 5]. Consistent with a women’s health perspective, qualitative evidence indicates that many women with vaginismus experience low awareness, stigma, and disempowering clinical encounters that can delay help-seeking and compromise care pathways [6].

A growing body of work suggests that avoidance in lifelong vaginismus may extend beyond penetration attempts and generalize to other sexual cues and non-penetrative sexual stimuli, for example avoidance of genital contact, nudity, bodily fluids (e.g., semen), and related sexual cues [1, 7–10]. Such cue-specific avoidance patterns may also shape clinical trajectories by influencing women’s willingness to disclose symptoms, tolerate examinations, and engage with stepwise treatment—domains highlighted as problematic in patient-reported help-seeking experiences [6]. These avoidance patterns are often conceptualized within a fear-avoidance framework, where anticipated pain-related fear and anxiety and catastrophizing may maintain avoidance via negative reinforcement [1, 11]. More recently, disgust has been highlighted as a key emotional mechanism that can motivate avoidance of sexual cues, particularly when genital contact or bodily fluids elicit contamination-related appraisals [8, 12]. From an affective–motivational perspective, disgust is theorized to promote withdrawal from cues appraised as contaminating and may contribute to avoidance by reinforcing short-term relief in response to those cues [13]. In vaginismus-related presentations, this account may be particularly relevant when avoidance targets non-penetrative sexual cues (e.g., genital contact, bodily fluids, nudity) rather than penetration attempts alone [14, 15]. Accordingly, specifying which cues are avoided may help characterize clinically relevant avoidance profiles even when current nosologies do not retain a discrete sexual aversion disorder category [12, 14]. Contemporary reviews and empirical studies on sexual aversion and disgust emphasize that disgust-driven avoidance may remain clinically meaningful even when a distinct sexual aversion disorder is not recognized in current diagnostic systems [8, 12].

Laboratory-based research using eye-tracking technology has provided empirical evidence for altered attentional processing of sexual cues among women with sexual difficulties. Experimental studies suggest that visual attention to sexual stimuli facilitates sexual arousal, whereas women with sexual dysfunction may allocate less attention to sexually relevant cues and more attention to threat-related information [16, 17]. For example, women with sexual dysfunction have been shown to attend less to genital regions in erotic stimuli than women with normal sexual functioning [17], and attentional biases toward sex-related threat cues have been proposed as factors contributing to impaired arousal and persistent avoidance in GPPPD and vaginismus [16]. These findings highlight the potential relevance of attentional processes in the maintenance of sexual difficulties and avoidance behaviors. These attentional patterns may be conceptually linked to metacognitive models emphasizing threat monitoring, hypervigilance, and self-focused attention [18–20].

Beyond affective mechanisms, cognitive processes may also shape maintenance of symptoms. Metacognition refers to beliefs about thinking (e.g., beliefs that worry is uncontrollable or dangerous) and the strategies used to regulate internal experiences [18, 19]. These include, for example, positive beliefs about the usefulness of worry (e.g., “Worrying helps me cope”), negative beliefs about the uncontrollability and danger of thoughts (e.g., “My worrying is out of control and harmful”), and beliefs about the need to control thoughts. In the present study, we focused specifically on dysfunctional metacognitive beliefs, referring to metacognitive beliefs that are thought to contribute to threat monitoring, worry, rumination, and maladaptive control strategies. Metacognitive theory posits that negative beliefs about the uncontrollability and danger of internal experiences increase threat monitoring and maladaptive control strategies (e.g., worry and rumination), thereby amplifying threat appraisals and contributing to distress and functional impairment [18–20]. In sexual contexts, such a syndrome may bias attention toward penetration-related threat cues (e.g., nudity, genital contact, bodily fluids) and promote avoidance as a short-term emotion-regulation strategy [13, 15, 18]. Dysfunctional metacognitive beliefs have been implicated across anxiety- and avoidance-related conditions and may influence how sexual cues are appraised and managed [18–21]. Systematic evidence suggests that dysfunctional metacognitive beliefs are associated with greater distress and impairment across sexual dysfunctions [21]. Given evidence that avoidance in lifelong vaginismus may generalize beyond penetration attempts to broader sexual cues, clarifying whether dysfunctional metacognitive beliefs relate to sexual functioning and cue-specific avoidance may refine assessment and case formulation [1, 7–10, 21]. Accordingly, we hypothesized that dysfunctional metacognitive beliefs may be more proximally linked to sexual functioning than to the mere presence of specific avoidance items. However, to our knowledge, few studies have examined dysfunctional metacognitive beliefs alongside clinician-elicited sex-related avoidance behaviors in women with lifelong vaginismus.

Therefore, in a cross-sectional case–control design, we examined clinician-elicited cue-specific avoidance behaviors together with dysfunctional metacognitive beliefs and sexual outcomes. Specifically, we aimed to address this gap by (1) characterizing the prevalence of clinician-elicited sex-related avoidance behaviors in women with lifelong vaginismus, (2) comparing sexual functioning and dysfunctional metacognitive beliefs across avoidant and non-avoidant vaginismus subgroups and a control group, and (3) examining whether dysfunctional metacognitive beliefs are associated with the avoidance subgroup membership and with sexual functioning above and beyond anxiety and depressive symptoms.

## Methods

### Design and Setting

This cross-sectional case–control study was carried out between 2019 and 2021 in three psychiatry outpatient clinics in Türkiye. The clinics were selected based on the availability of experienced psychiatrists with expertise in sexual dysfunction assessment and sexual therapy, as well as the feasibility of consecutive patient recruitment. Eligible patients presenting for evaluation of penetration-related sexual difficulties were approached consecutively during routine appointments; controls were recruited from healthy volunteers and screened for absence of a current sexual complaint. Ethical approval was obtained from the local ethics committee (2011-KAEK-25 2019/09–17). All participants provided written informed consent and completed study measures in a private setting. Questionnaire data were de-identified using unique study codes and stored securely to maintain confidentiality.

### Participants

Women with lifelong vaginismus were recruited consecutively from the participating clinics. Lifelong vaginismus was defined in routine clinical practice consistent with DSM-5 TR criteria for Genito-Pelvic Pain/Penetration Disorder, as primary penetration-related fear-avoidance accompanied by involuntary pelvic floor muscle tightening during attempted vaginal penetration (*n* = 64). In total, 72 eligible patients, aged ≥ 18 years and reporting a current heterosexual partner relationship, were approached; 64 were included in the final analyses, while two declined participation and six were excluded due to missing data. Eligibility criteria included age ≥ 18 years, a current heterosexual partner relationship, and sexual activity within the past 6 months. Thirty potential healthy controls were screened, and all were eligible and included (*n* = 30). Eligibility for controls required no current sexual complaints and no prior clinical consultation or admission due to a sexual problem. No financial compensation or other incentives were provided.

Exclusion criteria were applied based on clinical interview and medical history as documented in clinical records (e.g., severe medical or neurological illness that could affect sexual function, acute psychotic symptoms, or inability to complete self-report measures). Because a structured diagnostic interview was not administered, diagnostic eligibility and exclusions were determined by experienced clinicians using routine clinical assessment procedures in the participating clinics. All questionnaire data were recorded using unique study codes to preserve confidentiality.

### Assessment of sex-related avoidance behaviors

During routine clinical assessment (individual interview and, when applicable, couple interview), all participants, including controls, were asked standardized yes/no questions regarding sex-related avoidance behaviors over the past 6 months. The exact wording of the clinician-elicited avoidance questions (English translation) is provided in Supplementary Appendix A. Assessments were conducted by the same clinicians at each site as part of routine care, and responses were recorded as binary endorsements. The following behaviors were assessed: (1) avoidance of looking at genitals, (2) avoidance of touching one’s own genitals, (3) avoidance of touching the partner’s genitals, (4) avoidance of contact with semen or other bodily fluids, and (5) avoidance of nudity or remaining naked during sexual interaction. Participants endorsing ≥ 1 item were classified as the avoidant vaginismus subgroup (AV), whereas those endorsing none were classified as non-avoidant (Not-AV). This clinician-elicited checklist was used to characterize clinically salient avoidance patterns and was not intended as a validated psychometric scale; therefore, findings regarding avoidance subgroup membership should be interpreted as a clinician-elicited behavioral characterization rather than a measured latent construct [8, 12]. These items were selected to index clinically observable avoidance of sexual cues that are frequently described in aversion- and disgust-oriented formulations (e.g., nudity, genital contact, bodily fluids), thereby complementing penetration-focused symptoms. Because the checklist was used as a clinician-elicited characterization of avoidance (not a psychometric instrument), responses were coded dichotomously to maximize feasibility and consistency within routine clinical assessment. Clinician-elicited sex-related avoidance questions are provided in Supplementary Material.

### Measures

The Sociodemographic and Clinical Data Form, Beck Depression Inventory (BDI), Beck Anxiety Inventory (BAI), Arizona Sexual Experiences Scale (ASEX), Golombok–Rust Inventory of Sexual Satisfaction (GRISS), and Metacognitions Questionnaire-30 (MCQ-30) were administered to all participants. All questionnaires were completed in a private setting as self-report measures.

#### Sociodemographic and clinical data form

This form was developed by the researchers to assess participants’ sociodemographic characteristics (e.g., age, education level, relationship or marital duration, and type of acquaintanceship) and relevant clinical variables (e.g., sexual experiences before marriage and sex-related avoidance behaviors during sexual interaction). Avoidance behaviors were additionally evaluated using the clinician-elicited checklist described above.

#### Beck Depression Inventory (BDI)

It is developed by Beck et al. (1961) to assess the severity of depression symptoms in adults. Higher total scores indicate greater depressive symptom severity. A cut-off score of 17 has been suggested for clinically significant depressive symptoms [22]. The Turkish validity and reliability study was conducted in a Turkish sample [23].

#### Beck Anxiety Inventory (BAI)

It is a self-report measure of anxiety symptom severity and assesses the frequency of common anxiety symptoms and the extent to which they have bothered the individual over the past week. Higher total scores indicate greater anxiety symptom severity [24]. The Turkish version has demonstrated acceptable validity and reliability [25].

####  Metacognitions questionnaire-30 (MCQ-30)

It assesses metacognitive beliefs and consists of 30 items rated on a 4-point Likert scale from “strongly disagree” to “strongly agree”. The scale yields a total score and five subscales: positive beliefs about worry, negative beliefs about uncontrollability and danger, cognitive confidence, cognitive self-consciousness, and beliefs about the need to control thoughts. Higher scores indicate more dysfunctional metacognitive beliefs [19]. The Turkish version has demonstrated acceptable validity and reliability [26].

#### Arizona sexual experiences scale (ASEX)

The ASEX is a five-item questionnaire developed to provide a brief and reliable assessment of self-reported overall sexual functioning. The measure is available in sex-specific versions for women and men; the female version was administered in the present study. It assesses libido, psychological arousal, vaginal lubrication/penile erection, ability to reach orgasm, and orgasm satisfaction. Items are rated on a 6-point Likert scale (1 = optimal functioning, 6 = maximal impairment), yielding total scores from 5 to 30, with higher scores indicating greater sexual dysfunction [27]. The Turkish version has demonstrated acceptable validity and reliability [28].

#### Golombok-rust sexual satisfaction scale (GRISS)

The GRISS is a self-report measure developed to assess sexual functioning and satisfaction in heterosexual couples. It includes separate female and male forms; in this study, the female form was used. Both forms comprise seven subscales, five of which are shared (avoidance, dissatisfaction, non-communication, non-sensuality, and infrequency), while the female form additionally includes vaginismus and anorgasmia subscales. For the GRISS, subscale scores ≥ 5 were considered indicative of the corresponding sexual problem, with higher scores reflecting greater sexual dysfunction and lower sexual satisfaction [29]. The Turkish version has demonstrated acceptable validity and reliability [30].

The ASEX and GRISS were included because they assess complementary aspects of sexual functioning. Whereas the ASEX provides a brief measure of individual sexual functioning (e.g., desire, arousal, lubrication, orgasm, and satisfaction), the GRISS additionally evaluates broader dimensions of sexual satisfaction and relational functioning, including communication, avoidance, dissatisfaction, and frequency of sexual activity. The use of both instruments provided a more comprehensive assessment of sexual functioning, allowing examination of associations with metacognitive beliefs across both individual and relationship-related domains of sexual well-being.

### Statistical analysis

All statistical analyses were performed using IBM SPSS Statistics for Windows, Version 22.0 (IBM Corp., Armonk, NY, USA). Continuous variables were summarized as mean ± SD or median (IQR), as appropriate, and categorical variables as n (%). Between-group comparisons used chi-square or Fisher’s exact tests for categorical variables and one-way ANOVA or Kruskal–Wallis tests for continuous variables, as appropriate. When the overall test was significant, post-hoc pairwise comparisons were performed using Tukey’s HSD (after ANOVA) or Mann–Whitney U tests with Bonferroni adjustment (after Kruskal–Wallis). For post-hoc analyses, only adjusted significant pairwise differences were considered statistically significant. Within the vaginismus sample, binary logistic regression was conducted to examine associations with avoidance subgroup membership (AV vs. Not-AV), with BDI and BAI entered in Block 1 and three theoretically selected MCQ-30 subscales entered in Block 2. Hierarchical multiple regression analyses were conducted to examine whether MCQ-30 subscales accounted for additional variance in ASEX-total and GRISS-total scores above and beyond BDI and BAI symptom levels. In these models, BDI and BAI were entered in Step 1, and MCQ-30 subscale scores were entered simultaneously in Step 2. Anxiety and depressive symptom levels were included as covariates to examine whether metacognitive beliefs were associated with sexual outcomes beyond general distress. The incremental explanatory value of metacognitive beliefs was evaluated by examining the change in explained variance (ΔR²) from Step 1 to Step 2. The significance level was set at *p* < 0.05 for all analyses.

## Results

### Sample characteristics

The study included 64 women with lifelong vaginismus and 30 controls. Within the vaginismus sample, 30 participants (46.9%) affirmed at least one clinician-elicited sex-related avoidance item and were classified as avoidant (AV), while 34 (53.1%) affirmed none and were classified as non-avoidant (Not-AV). As shown in Table 1, mean age did not differ significantly across the vaginismus subgroups and controls. The groups were otherwise comparable on key sociodemographic characteristics (Table 1).

**Table 1:** Sociodemographic and clinical characteristics of women with lifelong vaginismus by avoidance subgroup (AV vs. Not-AV) and controls

	AV group (*n* = 30)	Not-AV group (*n* = 34)	Control group (*n* = 30)	*p*
Age (Mean/SD)	26.97 (4.26)	25.59 (4.19)	27.07 (3.68)	0.264
Duration of marriage (Mean/SD)	19.97 (18.16)	17.12 (28.22)	14.07 (15.38)	0.141
Education level (n/%)				0.050
* ≤8 years of education*	12 (40.0)	7 (20.5)	7 (23.3)	
* High school*	9 (30.0)	11 (32.4)	17 (56.7)	
* University*	9 (30.0)	16 (47.1)	6 (20.0)	
Type of acquaintanceship (n/%)				0.626
* By dating*	24 (80.0)	30 (88.2)	26 (86.7)	
* By matchmaking*	6 (20.0)	4 (11.8)	4 (13.3)	
Sexual experiences before marriage (n/%)				0.217
* None*	20 (66.7)	15 (44.1)	17 (56.7)	
* Only kissing*	5 (16.7)	8 (23.5)	9 (30.0)	
* Clothed foreplay*	5 (16.7)	11 (32.4)	4 (13.3)	

Values are presented as n (%) for categorical variables and as mean/SD for continuous variables, SD: Standard Deviation, AV = Avoidant Vaginismus subgroup, Not-AV = Non-Avoidant vaginismus subgroup, A two-tailed *p*< 0.05 was considered statistically significant

### Prevalence of sex-related avoidance behaviors

Thirty of 64 women with lifelong vaginismus (46.9%) endorsed at least one avoidance behavior, whereas none of the control participants did (*p* < 0.001). Within the AV subgroup, avoidance was most often limited to a single behavior: 20 participants (66.7%) affirmed one behavior, whereas 5 (16.7%) affirmed two behaviors, 4 (13.3%) affirmed three behaviors, and 1 (3.3%) affirmed four behaviors. These findings indicate that avoidance of sexual cues beyond penetration attempts was present in nearly half of women with lifelong vaginismus, although multiple concurrent avoidance behaviors were relatively uncommon (Table 2).

**Table 2:** Item-level prevalence of clinician-elicited sex-related avoidance behaviors (past 6 months)

Clinician-elicited avoidance item (Yes/No)	Lifelong vaginismus total (*n* = 64), n (%)	AV subgroup (*n* = 30), *n* (%)
Avoided touching her own genitals	7 (10.9)	7 (23.3)
Avoided touching partner’s penis	10 (15.6)	10 (33.3)
Avoided touching semen	19 (29.7)	19 (63.3)
Avoided having sex with the lights on	5 (7.8)	5 (16.7)
Avoided being naked during foreplay	5 (7.8)	5 (16.7)

AV was defined as endorsing ≥ 1 avoidance item; Not-AV was defined as endorsing none. Therefore, item-level frequencies in the Not-AV subgroup are 0% by definition and are not shown; counts in the total vaginismus sample equal the AV counts, while percentages differ because of the denominator

### Group differences in depressive and anxiety symptoms and dysfunctional metacognitive beliefs

Pairwise comparisons showed that BAI scores did not differ significantly across AV, Not-AV, and control groups. BDI scores were significantly higher in AV than controls, whereas BDI did not differ between AV and Not-AV groups. In contrast, MCQ-30 total scores and the MCQ-30 subscales of negative beliefs about uncontrollability and danger, and beliefs about the need to control thoughts were significantly higher in both vaginismus subgroups than in controls. However, the AV and Not-AV vaginismus groups did not differ significantly in overall dysfunctional metacognitive beliefs or any MCQ-30 subscale scores (all *p* > 0.05) (Table 3).

**Table 3:** Between-group comparisons of depressive and anxiety symptoms, metacognitive beliefs, and sexual functioning in avoidant and non-avoidant lifelong vaginismus and control groups

	AV group (*n* = 30)	Not-AV group (*n* = 34)	Control group (*n* = 30)	Overall *p*	Effect size (Hedges’ g)
	Mean (SD)	Mean (SD)	Mean (SD)		
BDI	12.47 (7.03)^a^	10.56 (6.05)^ab^	8.40 (3.74)^b^	0.028	
BAI	11.97 (6.10)^a^	14.21 (9.52)^a^	9.87 (3.94)^a^	0.053	
MCQ-30-Total	75.47 (13.49)^a^	72.53 (13.71)^a^	62.00 (8.75)^b^	< 0.001	
MCQ-30-positive beliefs about worry	11.67 (3.64)^a^	10.35 (3.29)^ab^	9.40 (2.95)^b^	0.032	
MCQ-30-negative beliefs about uncontrollability of thoughts and danger	18.23 (5.62)^a^	17.12 (4.62)^a^	13.93 (3.22)^b^	0.001	
MCQ-30-cognitive confidence	13.43 (5.22)^a^	13.03 (4.56)^a^	11.27 (3.89)^a^	0.155	
MCQ-30-cognitive self-consciousness	17.87 (3.73)^a^	17.62 (3.63)^a^	16.50 (2.54)^a^	0.244	
MCQ-30-beliefs about need to control thoughts	14.27 (3.80)^a^	14.44 (5.06)^a^	10.90 (2.58)^b^	< 0.001	
GRISS-Total	58.20 (17.76)^a^	44.35 (12.97)^b^	32.03 (10.76)^c^	< 0.001	AV vs. Not-AV, 0.89; AV vs. Control, 1.76; Not-AV vs. Control, 1.02
GRISS-infrequency	4.67 (2.19)^a^	3.59 (2.20)^ab^	3.23 (1.72)^b^	0.021	
GRISS-non-communication	4.70 (2.28)^a^	2.74 (1.94)^b^	2.70 (1.86)^b^	< 0.001	
GRISS-avoidance	7.13 (3.56)^a^	4.18 (3.48)^b^	3.83 (2.35)^b^	< 0.001	
GRISS-non-sensuality	6.23 (3.72)^a^	3.09 (2.27)^b^	3.20 (2.17)^b^	< 0.001	
GRISS-dissatisfaction	6.07 (2.85)^a^	5.79 (2.77)^a^	4.17 (2.53)^b^	0.016	
GRISS-vaginismus	11.87 (2.69)^a^	12.35 (2.44)^a^	5.00 (2.17)^b^	< 0.001	
GRISS-anorgasmia	9.43 (4.22)^a^	6.62 (3.44)^b^	5.03 (2.51)^b^	< 0.001	
ASEX-Total	19.47 (6.50)^a^	12.53 (4.37)^b^	10.07 (2.84)^c^	< 0.001	AV vs. Not-AV, 1.25; AV vs. Control, 1.85; Not-AV vs. Control, 0.65
ASEX- drive	3.80 (1.45)^a^	2.35 (1.32)^b^	2.00 (0.87)^b^	< 0.001	
ASEX- arousal	4.17 (1.37)^a^	2.68 (1.07)^b^	2.07 (0.83)^c^	< 0.001	
ASEX- lubrication/erection	3.80 (1.32)^a^	2.32 (1.07)^b^	1.83 (0.75)^b^	< 0.001	
ASEX- orgasm	4.03 (1.40)^a^	2.79 (1.07)^b^	2.20 (0.81)^c^	< 0.001	
ASEX- satisfaction	3.63 (1.71)^a^	2.38 (1.16)^b^	1.97 (0.85)^b^	< 0.001	

Values are presented as mean (SD). Within each row, values with different superscript letters differ significantly in adjusted pairwise comparisons; values sharing at least one superscript letter do not differ significantly. Overall *p* values were obtained using one-way ANOVA or Kruskal–Wallis tests, as appropriate. Effect sizes are reported as Hedges’ g for the primary sexual outcome measures (GRISS total and ASEX total). SD: Standard Deviation, AV: Avoidant Vaginismus, Not-AV: Non-Avoidant Vaginismus, BDI: Beck Depression Inventory, BAI: Beck Anxiety Inventory, MCQ-30: Metacognitions Questionnaire-30, GRISS: Golombok-Rust Sexual Satisfaction Scale, ASEX: Arizona Sexual Experiences Scale

### Group differences in sexual outcomes

Sexual outcomes showed a robust graded pattern across groups. The AV subgroup demonstrated significantly poorer sexual outcomes than Not-AV, with higher GRISS-Total scores and higher scores on the GRISS non-communication, avoidance, and anorgasmia subscales, as well as higher ASEX-Total and higher scores across all ASEX subscales (all *p* < 0.05). Relative to controls, Not-AV participants also showed worse sexual outcomes, including higher GRISS-Total (with significant differences in dissatisfaction and vaginismus subscales) and higher ASEX-Total (with significant differences in arousal and ability to reach orgasm) (all *p* < 0.05). Compared with controls, AV participants had significantly higher scores on both total and subscale scores of GRISS and ASEX (all *p* < 0.05) (Table 3).

### Dysfunctional metacognitive beliefs and avoidance subgroups (AV vs. Not-AV)

Within the vaginismus sample (*n* = 64), binary logistic regression was conducted to examine associations with AV subgroup membership. In Block 1, BDI and BAI were entered as covariates; in Block 2, three theoretically selected MCQ-30 subscales (positive beliefs about worry, negative beliefs about uncontrollability and danger, and beliefs about the need to control thoughts) were added. The final model was statistically significant overall (χ²(5) = 11.54, *p* = 0.042; Nagelkerke R²=0.22; classification accuracy = 68.8%). However, adding the three MCQ-30 subscales did not significantly improve model fit beyond BDI and BAI (Δχ²(3) = 3.84, *p* = 0.279). In the final model, none of the three MCQ-30 subscales showed a significant independent association with AV subgroup membership, whereas higher BDI scores were positively associated and higher BAI scores were negatively associated with AV status (Table 4).

**Table 4:** Binary logistic regression examining associations with avoidance subgroup membership (AV vs. Not-AV) within the vaginismus sample

	B	SE	Wald	*p*	Exp(B)	95% CI	
						Lower	Upper
BDI	0.175	0.070	6.147	0.013	1.191	1.037	1.367
BAI	-0.123	0.053	5.324	0.021	0.885	0.797	0.982
MCQ-30-positive beliefs about worry	0.107	0.084	1.634	0.201	1.113	0.944	1.312
MCQ-30-negative beliefs about uncontrollability of thoughts and danger	0.043	0.059	0.539	0.463	1.044	0.930	1.172
MCQ-30-beliefs about need to control thoughts	-0.065	0.071	0.830	0.362	0.937	0.815	1.077
Constant	-1.541	1.546	0.994	0.319	0.214	0.010	4.430

Binary logistic regression was conducted within the vaginismus sample (*n* = 64) to examine associations with avoidance subgroup membership (AV vs. Not-AV). In Block 1, BDI and BAI scores were entered as covariates; in Block 2, three theoretically selected MCQ-30 subscales (positive beliefs about worry, negative beliefs about uncontrollability and danger, and beliefs about the need to control thoughts) were entered simultaneously. Results are presented as regression coefficients (B), standard errors (SE), Wald statistics, odds ratios [Exp(B)], 95% confidence intervals (CI), and p values. The final model was statistically significant overall (χ²(5) = 11.54, *p* = 0.042; Nagelkerke R²=0.22; classification accuracy = 68.8%), but the addition of the three MCQ-30 subscales did not significantly improve model fit beyond BDI and BAI (Δχ²(3) = 3.84, *p* = 0.279). AV: avoidant vaginismus; Not-AV: non-avoidant vaginismus; BDI: Beck Depression Inventory; BAI: Beck Anxiety Inventory; MCQ-30: Metacognitions Questionnaire-30

### Dysfunctional metacognitive beliefs and sexual outcomes beyond anxiety and depression

Hierarchical multiple regression analyses examined whether MCQ-30 subscales accounted for variance in sexual outcomes beyond depressive and anxiety symptoms (Table 5). For ASEX-Total, BDI and BAI scores explained 13% of the variance in Step 1, and adding MCQ-30 subscale scores accounted for an additional 19% of variance in Step 2 (total R²=0.32; ΔR²=0.19). In the final model, positive beliefs about worry and negative beliefs about uncontrollability and danger were associated with higher ASEX-Total scores, whereas cognitive self-consciousness was inversely associated with ASEX-Total scores. For GRISS-Total, BDI and BAI scores explained 5% of the variance in Step 1, and adding MCQ-30 subscale scores accounted for an additional 21% of variance in Step 2 (total R²=0.26; ΔR²=0.21). In the final model, negative beliefs about uncontrollability and danger remained positively associated with higher GRISS-Total scores, whereas positive beliefs about worry did not show a significant independent association with GRISS-Total scores.

**Table 5:** Hierarchical regression models examining associations of metacognitive beliefs with ASEX-Total and GRISS-Total scores beyond depressive and anxiety symptoms

		*R* ^2^	B	SE	β	t	*p*
		ASEX-Total					
Step 1	(Constant)	0.13*	12.26	1.63		7.51	< 0.001
	BDI		0.42	0.15	0.43	2.77	0.007
	BAI		-0.10	0.12	-0.12	-0.80	0.426
Step 2	(Constant)	0.32**	10.96	4.43		2.47	0.017
	BDI		0.44	0.15	0.44	2.90	0.005
	BAI		-0.05	0.12	-0.06	-0.43	0.666
	MCQ-30-positive beliefs about worry		0.49	0.22	0.26	2.22	0.030
	MCQ-30-negative beliefs about uncontrollability of thoughts and danger		0.66	0.22	0.52	3.02	0.004
	MCQ-30-cognitive confidence		-0.17	0.17	-0.13	-1.01	0.317
	MCQ-30-cognitive self-consciousness		-0.87	0.28	-0.49	-3.13	0.003
	MCQ-30-beliefs about need to control thoughts		0.09	0.18	0.06	0.50	0.616
		GRISS-Total					
Step 1	(Constant)	0.05	44.24	4.43		9.98	< 0.001
	BDI		0.64	0.41	0.25	1.55	0.126
	BAI		-0.05	0.33	-0.03	-0.16	0.872
Step 2	(Constant)	0.26*	30.80	12.01		2.57	0.013
	BDI		0.72	0.41	0.28	1.77	0.083
	BAI		0.05	0.31	0.02	0.16	0.872
	MCQ-30-positive beliefs about worry		0.45	0.59	0.09	0.75	0.454
	MCQ-30-negative beliefs about uncontrollability of thoughts and danger		1.92	0.59	0.58	3.27	0.002
	MCQ-30-cognitive confidence		0.15	0.46	0.04	0.32	0.751
	MCQ-30-cognitive self-consciousness		-1.85	0.75	-0.40	-2.46	0.017
	MCQ-30-beliefs about need to control thoughts		0.22	0.49	0.59	0.46	0.651

*<0.05, **<0.01, *n* = 64. Hierarchical multiple regression models were used to examine whether metacognitive beliefs (MCQ-30 total or subscales, as specified) explained variance in sexual outcomes beyond anxiety and depressive symptoms. Values are presented as unstandardized coefficients (B), standard errors (SE), standardized coefficients (β), t statistics, and p values. In Step 1, BDI and BAI were entered; in Step 2, MCQ-30 subscale scores were added simultaneously. For ASEX-Total, Step 1 R²=0.13 and Step 2 R²=0.32 (ΔR²=0.19). For GRISS-Total, Step 1 R²=0.05 and Step 2 R²=0.26 (ΔR²=0.21). ASEX: Arizona Sexual Experiences Scale (female form); GRISS: Golombok–Rust Inventory of Sexual Satisfaction (female form); BDI: Beck Depression Inventory; BAI: Beck Anxiety Inventory; MCQ-30: Metacognitions Questionnaire-30

## Discussion

### Principal findings

The present study makes three contributions to the understanding of lifelong vaginismus. First, we demonstrate that cue-specific avoidance beyond penetration attempts is present in nearly half of women with lifelong vaginismus and is entirely absent in controls, supporting the clinical relevance of assessing avoidance behaviors that extend beyond the defining symptom of penetration difficulty. Second, avoidant vaginismus was associated with markedly poorer sexual functioning and satisfaction relative to both non-avoidant vaginismus and controls, indicating that avoidance subtyping captures meaningful clinical heterogeneity within this presentation. Third, metacognitive beliefs—particularly negative beliefs about uncontrollability and danger—were independently associated with sexual impairment after adjusting for depressive and anxiety symptoms, extending prior evidence linking dysfunctional metacognition to sexual distress and suggesting that cognitive maintenance processes may contribute to sexual impairment in lifelong vaginismus above and beyond general negative affect.

### Findings in the context of disgust- and avoidance-based models

The high prevalence of avoidance behaviors in vaginismus is consistent with models emphasizing avoidance as a maintaining factor [1, 31, 32]. In the present study, avoidance was captured via clinician-elicited yes/no items assessing avoidance of sexual cues beyond penetration attempts, which aligns with contemporary frameworks emphasizing the clinical relevance of cue-specific aversion and avoidance processes [7, 8, 10, 12].

In line with these accounts, the avoidant subgroup (AV) showed substantially poorer sexual outcomes than the non-avoidant subgroup (Not-AV), particularly on global indices (ASEX-Total; GRISS-Total) and on GRISS domains reflecting avoidance and relational functioning (e.g., avoidance and non-communication). These between-subgroup differences were observed despite the absence of subgroup differences in depressive and anxiety symptoms (BDI, BAI) and MCQ-30 indices, suggesting that broader sexual-cue avoidance may capture clinically meaningful heterogeneity within lifelong vaginismus presentations [8, 14].

Recent work has questioned whether sexual aversion is best conceptualized as a discrete diagnostic entity and has instead proposed aversion-related symptoms as transdiagnostic dimensions across sexual dysfunction presentations [8]. This perspective supports the value of specifying avoided sexual cues when characterising clinical subgroups within vaginismus [14]. Emerging intervention work, including hierarchical exposure-based approaches and virtual reality–supported exposure procedures, suggests potential benefit for directly targeting avoidance responses when planning treatment [9, 12].

Beyond clinical outcomes, sociodemographic trends observed in the present study may also provide insight into the heterogeneity of avoidance patterns. Notably, women in the avoidant subgroup tended to have lower educational attainment than those in the non-avoidant subgroup, although this association was at the threshold of statistical significance (*p* = 0.050). While this finding should be interpreted cautiously, it raises the possibility that educational and sociocultural factors may contribute to the expression of broader sexual avoidance. Lower educational attainment may be associated with differences in health literacy and access to sexual health information, factors that could potentially influence how sexual difficulties are understood and managed. Consistent with this interpretation, a cross-sectional study among married women found that health literacy was significantly associated with sexual functioning across multiple domains, and that educational attainment was among the key determinants of health literacy [33]. Educational background may therefore influence awareness of sexual health, willingness to seek help, and engagement with treatment. However, the present study was not designed to examine these mechanisms directly, and no causal conclusions can be drawn. Future studies with larger samples should further investigate the potential role of educational background, health literacy, and related sociocultural factors in shaping avoidance patterns among women with lifelong vaginismus.

### Dysfunctional metacognitive beliefs and sexual outcomes

In the present study, women with lifelong vaginismus reported greater endorsement of dysfunctional metacognitive beliefs than controls. This pattern is consistent with prior evidence linking dysfunctional metacognitive beliefs to sexual distress and related impairment [34, 35]. In the model for ASEX-Total, both positive beliefs about worry and negative beliefs about uncontrollability and danger were independently associated with poorer sexual outcomes above and beyond depressive and anxiety symptoms. In contrast, cognitive self-consciousness was inversely associated with ASEX outcomes. In the corresponding model for GRISS-Total, negative beliefs about uncontrollability and danger remained significantly associated with poorer sexual outcomes, whereas positive beliefs about worry were not. This pattern suggests that worry-related metacognitive beliefs may be more closely linked to individual aspects of sexual functioning assessed by the ASEX than to the broader dimensions of sexual satisfaction and relational functioning captured by the GRISS. One possible explanation is that beliefs regarding the usefulness of worry may promote heightened self-focused attention and monitoring of bodily sensations during sexual activity, processes that could more directly affect desire, arousal, and orgasmic functioning. However, because the present study was not designed to directly compare these associations across different sexual functioning and satisfaction outcomes, this interpretation should be considered tentative and warrants further investigation. Within metacognitive theory, such beliefs are proposed to promote worry as a coping strategy and thereby sustain cognitive attentional syndrome processes such as threat monitoring and self-focused attention [18–20]. In sexual contexts, endorsing a “worry-is-useful” stance may translate into heightened anticipatory worry and hypervigilance to bodily cues (e.g., pain, muscle tension, arousal), which can compete with attention to erotic cues and contribute to impaired arousal and orgasmic responding [18–20, 32]. These findings are broadly consistent with previous work reporting associations between metacognitive beliefs and sexual functioning [36]. Our findings may also be considered in relation to experimental research on attentional processing of sexual cues. Eye-tracking studies have reported that women with sexual difficulties attend less to sexually relevant stimuli and may exhibit greater attention to threat-related information [16, 17]. Although attentional processes were not directly assessed in the present study, the observed associations between dysfunctional metacognitive beliefs and poorer sexual outcomes are conceptually consistent with models proposing that threat monitoring and self-focused attention interfere with adaptive sexual responding. In particular, negative beliefs about the uncontrollability and danger of internal experiences may contribute to the maintenance of hypervigilance toward threat-related cues, thereby reducing engagement with sexually relevant stimuli. Future studies combining metacognitive assessments with experimental measures of attentional allocation may help clarify these mechanisms in women with lifelong vaginismus. In metacognitive models, negative metacognitive beliefs are theorized to amplify threat appraisals and maintain distress via the cognitive attentional syndrome (e.g., threat monitoring, worry, rumination, and maladaptive thought-control strategies) [18–20]. In the context of lifelong vaginismus, heightened monitoring of penetration-relevant cues and situations (e.g., nudity or genital contact) may be experienced as threatening and may co-occur with broader avoidance patterns. The inverse association between cognitive self-consciousness and sexual outcomes should be interpreted cautiously, as the direction of association may vary by context and measurement; replication using more fine-grained process measures and prospective designs is warranted.

### Clinical implications

Patient-reported work emphasizes that improving vaginismus care requires greater awareness, destigmatization, and patient empowerment during clinical encounters [6]. Aligned with this patient-centered agenda, the present results add a complementary clinical assessment perspective. Cue-specific avoidance was common in our sample and was associated with substantially poorer sexual outcomes, supporting routine cue-focused screening in practice. Accordingly, women presenting with lifelong vaginismus-related complaints may benefit from a structured assessment of sex-related avoidance beyond penetration attempts. Given the high proportion of participants reporting at least one clinician-elicited avoidance behavior, cue-focused screening appears feasible in routine care. It may also help identify individuals with broader avoidance repertoires and greater impairment.

Disgust-related avoidance processes may be clinically meaningful even when not represented as a discrete diagnostic category and may be conceptualized as transdiagnostic phenomena across sexual dysfunctions [8, 13, 15]. From this perspective, specifying which sexual cues are avoided—rather than treating avoidance as a unitary construct—can refine case formulation and highlight individualized intervention targets [8]. Complementing this approach, metacognitive models posit that dysfunctional metacognitive beliefs (especially negative beliefs about uncontrollability and danger) can promote threat monitoring and avoidance [37]. In our data, negative metacognitive beliefs were associated with poorer sexual outcomes above and beyond depressive and anxiety symptoms. This supports incorporating the assessment of dysfunctional metacognitive beliefs into individualized formulations, even when metacognitive beliefs do not differentiate avoidance subgroups.

From a treatment-planning standpoint, interventions that directly address avoidance responses may be particularly relevant for patients with broad cue avoidance. These most commonly include graded, systematic exposure to feared and disgust-eliciting sexual cues while preventing avoidance [9, 12]. Technology-assisted formats such as virtual reality exposure therapy represent an emerging option with potential acceptability and clinical benefit in aversion-related sexual difficulties [8, 9, 14].

Notably, negative metacognitive beliefs about uncontrollability and danger remained associated with poorer sexual outcomes even after accounting for depressive and anxiety symptom levels, showing significant associations with higher ASEX-Total and GRISS-Total scores in hierarchical models. This pattern supports considering adjunctive metacognitive or cognitive-behavioral strategies that target threat appraisals and downstream “cognitive attentional syndrome” processes (e.g., threat monitoring, worry, rumination, and dysfunctional thought-control efforts) [37]. Such work may be particularly relevant for patients with marked sexual impairment, where internal threat-related beliefs may perpetuate distress and hinder engagement in corrective sexual experiences. Finally, these treatment implications should be framed as hypothesis-generating given the cross-sectional design, and the inverse association observed for cognitive self-consciousness warrants replication.

### Strengths and limitations

Strengths include the case–control design, the clinician-elicited characterization of sex-related avoidance behaviors in women with lifelong vaginismus, and the integration of dysfunctional metacognitive beliefs with sexual outcomes. Limitations include the modest sample size and the cross-sectional design, which precludes causal inference. Because participants were recruited from treatment-seeking clinical settings, the findings may not fully generalize to community samples. A structured diagnostic interview was not administered; diagnostic and exclusion decisions relied on standard clinical evaluation by experienced clinicians. Finally, avoidance subgrouping was based on five dichotomous items, which may have reduced sensitivity to gradations in avoidance (e.g., frequency and intensity) and, in turn, weakened observed associations with cognitive variables. Given that avoidance in vaginismus is likely shaped by multiple determinants (e.g., fear-avoidance and disgust-related mechanisms alongside relational and contextual factors), future studies should use validated, multidimensional avoidance measures and larger samples to model these processes more comprehensively.

## Conclusion

Sex-related cue avoidance beyond penetration attempts was present in nearly half of women with lifelong vaginismus and was associated with markedly poorer sexual outcomes, with the avoidant subgroup showing the greatest impairment. Negative metacognitive beliefs—particularly beliefs about uncontrollability and danger—were associated with sexual impairment above and beyond depressive and anxiety symptoms. Accordingly, incorporating routine screening for cue-specific avoidance and relevant cognitive processes may support a more patient-centered assessment and facilitate engagement with treatment. Overall, these findings support routine cue-focused assessment and provide a rationale for prospective trials testing whether targeting avoidance (e.g., graded exposure, including technology-assisted formats) alongside dysfunctional metacognitive processes improves outcomes in lifelong vaginismus.

## Supplementary Information

Supplementary Material 1.

## Data Availability

Data are available from the corresponding author upon reasonable request.
