# Supplementary material for: Cue-specific sexual avoidance, metacognitive beliefs, and sexual functioning in women with lifelong vaginismus: a cross-sectional case–control study
Source: BMC Womens Health. 2026 Aug 3;26:449. doi: 10.1186/s12905-026-04761-z (PMC13560228; doi:10.1186/s12905-026-04761-z)
Supplement: Supplementary file 1 [file 12905_2026_4761_MOESM1_ESM.docx]

**SUPPLEMENTARY MATERIAL**

Supplementary Information for: Sex-Related Avoidance Behaviors, Metacognitive Beliefs, and Sexual Functioning in Women with Lifelong Vaginismus: A Cross-Sectional Case–Control Study

1. **Exact wording of clinician-elicited sex-related avoidance questions**

As part of routine clinical assessment (individual interview and, when applicable, couple interview), participants were asked the following standardized **yes/no** questions referring to the **past 6 months**. The questions were administered in Turkish in the clinics; the wording below represents the English translation used for reporting. Responses were recorded as binary endorsements.

**Clinician-elicited sex-related avoidance questions (Yes/No):**

1. In the past 6 months, have you avoided **looking at your genitals** (vulva)? *(Yes/No)*
2. In the past 6 months, have you avoided **touching your own genitals** (vulva)? *(Yes/No)*
3. In the past 6 months, have you avoided **touching your partner’s genitals?** *(Yes/No)*
4. In the past 6 months, have you avoided **contact with semen or other sexual bodily fluids** (e.g., genital secretions)? *(Yes/No)*
5. In the past 6 months, have you avoided **nudity or remaining naked during sexual interaction** (e.g., keeping clothing/underwear on during sexual activity)? *(Yes/No)*

**Classification rule (used in analyses):** Participants endorsing ≥1 item were classified as **Avoidant subgroup(AV);** those endorsing none were classified as **Non-Avoidant subgroup(Not-AV).**
